# Do Substance Use Risk Personality Dimensions Predict the Onset of Substance Use in Early Adolescence? A Variable- and Person-Centered Approach

**DOI:** 10.1007/s10964-012-9775-6

**Published:** 2012-05-24

**Authors:** Monique Malmberg, Marloes Kleinjan, Ad A. Vermulst, Geertjan Overbeek, Karin Monshouwer, Jeroen Lammers, Rutger C. M. E. Engels

**Affiliations:** 1 Behavioural Science Institute, Radboud University Nijmegen, P.O. Box 9104, 6500 HE Nijmegen, The Netherlands; 2 Developmental Psychology, Utrecht University, Utrecht, The Netherlands; 3 Trimbos Institute (Netherlands Institute of Mental Health and Addiction), Utrecht, The Netherlands; 4 Department of Interdisciplinary Social Science, Utrecht University, Utrecht, The Netherlands

**Keywords:** Alcohol, Tobacco, Marijuana, Personality, Early adolescence

## Abstract

Various studies found personality to be related to substance use, but little attention is paid to the role of personality risk dimensions with regard to an early onset of alcohol, tobacco, and marijuana use. Therefore, the current study used a variable-centered approach to examine whether anxiety sensitivity, hopelessness, sensation seeking, and impulsivity predict the onset of alcohol, tobacco, and marijuana use in early adolescence. Additionally, we adopted a person-centered approach to examine whether different personality subgroups could be identified, and whether these subgroups would be predictive of substance use. For that purpose, longitudinal data of a broader effectiveness study were used from 758 early adolescents (53 % female) aged 11–14 years. Structural equation models showed that hopelessness and sensation seeking were predictive of having ever used alcohol and tobacco. Also, sensation seeking was predictive of marijuana use. Latent profile analyses on the first wave data revealed a three-profile solution for boys (i.e., resilients, internalizers, and externalizers) and a two-profile solution for girls (i.e., resilients and internalizers). In contrast to our expectation, further analyses revealed no significant differences in substance use between the different subprofiles for both boys and girls. The separate personality dimensions thus seem more relevant in predicting the onset of substance use compared to the personality profiles. However, the personality profiles might be informative in explaining more excessive substance use behaviors.

## Introduction

Many Dutch adolescents start using alcohol, tobacco, and marijuana in their early teens (Hibell et al. 2009; Monshouwer et al. 2008). Forty-six percent of 12-year-old boys and 36 % of 12-year-old girls already report alcohol consumption (Van Dorsselaer et al. 2010). At age 12, approximately 12 % of Dutch adolescents have smoked at least once, increasing to 44 % at age 13-14 (Stivoro 2010), and 2.3 % of the 12-year-olds and over 10 % of the 14-year-olds report ever having used marijuana (Van Laar et al. 2010). Early substance use has many detrimental consequences, amongst which distortion of brain development (e.g., Tapert et al. 2002) and elevated risk for later substance dependence and misuse (e.g., Andersen et al. 2003; DiFranza et al. 2000). Given these adverse health effects, it is crucial to identify risk profiles of early adolescents, since this might facilitate adequate prevention efforts targeted at youths who are at risk for an early onset of substance use or abuse (e.g., Conrod et al. 2008; 2010). Insofar research has focused on individual factors in explaining adolescents’ substance use, most studies have focused on early pubertal timing—mostly in girls (Richards and Oinonen 2011; Stattin et al. 2011). Whereas the role of personality in alcohol, tobacco, and marijuana use has been well-established among already using groups (e.g., Chassin et al. 2002; Colder et al. 2002; Flory et al. 2002; Jackson et al. 2000; Loukas et al. 2000; Otten et al. 2008), relatively little research effort has gone into the examination of personality characteristics that might play a role in the onset of substance use in adolescence.

Personality is often defined as “individual differences in the tendency to behave, think, and feel in certain consistent ways” (Caspi 1998, p. 312) and these individual differences are argued to be relatively stable over time, due to biological origins as temperament (Asendorf and Denissen 2006; Eisenberg et al. 2000; Shiner 1998). As described in Malmberg et al. 2010b, specific personality dimensions concerning neurotic tendencies and deficits in behavioral inhibition are strong predictors of substance (mis)use (e.g., Barrett et al. 1998; Cloninger 1998). One instrument that specifically taps such dimensions is the Substance Use Risk Profile Scale (SURPS; Malmberg et al. 2010b; Woicik et al. 2009). This scale measures four distinct and independent personality dimensions, which are anxiety sensitivity, hopelessness, sensation seeking, and impulsivity. The anxiety sensitivity dimension is characterized by the fear of symptoms of psychical arousal (Reis et al. 1986), whereas the hopelessness dimension is identified as a risk factor for the development of depression and characterized by dismal feelings (Joiner 2001). The sensation seeking dimension is characterized by the desire for intense and novel experiences (Zuckerman 1994) and finally the impulsivity dimension involves difficulties in the regulation (controlling) of behavioral responses (Spoont 1992). Anxiety sensitivity, hopelessness, sensation seeking, and impulsivity are all personality risk factors that previously have been linked to alcohol misuse. The personality dimensions marking a broad impulsive sensation seeking trait are robust predictors of heavy alcohol use and alcohol use disorders. The neurotic personality traits also have shown to predict progression from adolescent drinking to alcohol problems in young adulthood (Conrod et al. 2006; Woicik et al. 2009). Conclusively, the four SURPS personality dimensions are not only hypothesized, but also found to relate to high and problematic substance use behaviors.

Although the SURPS personality dimensions demonstrated their usefulness in samples that already were using substances (Conrod et al. 1998; Jackson and Sher 2003; Pulkkinen and Pitkänen 1994; Shall et al. 1992; Sher et al. 2000; Stewart et al. 1995), little attention has been paid to the role of these personality dimensions with regard to the *early* onset of alcohol, tobacco, and marijuana use (Krank et al. 2011; Malmberg et al. 2010b). This is unfortunate, considering that early onset is one of the strongest identified risk factors for substance use problems in later life (Breslau et al. 1993; Chen et al. 2005; De Wit et al. 2000) and these personality predispositions may play a particularly important role in explaining risk behavior and receptivity for substance use during the period of adolescence (e.g., Carver et al. 2009; Malmberg et al. 2010b). One study prospectively investigated the role of the SURPS personality dimensions on early adolescent substance use and found hopelessness, sensation seeking, and impulsivity to be predictive of substance use behaviors 1 year later (Krank et al. 2011). However, this study controlled for prior substance use in their analyses without differentiating between never- and ever-users. One might argue not only that the personality dimensions influence substance use, but that substance use also modifies brain structures and possible associated personality predispositions (Carver et al. 2009; Graves et al. 2005; Tapert et al. 2002). In order to capture the “pure” predictive validity of the personality dimensions on substance use, prospective analyses in a never-using group of early adolescents is warranted.

Another limitation of prior research on the SURPS personality dimensions is the exclusive adherence to a variable-centered approach (e.g., Conrod et al. 2000; Ismail et al. 2009; Jaffee and D’Zurilla 2009; Krank et al. 2011; Siu 2010). A variable-centered approach focuses on differences among individuals on variables (Dubas et al. 2002) or on associations between predictor variables (i.e., SURPS personality dimensions) and outcome variables (i.e., substance use). Recently, scholars have argued that combining a person-centered approach with the variable-centered approach leads to a better understanding of processes and patterns underlying human behavior (e.g., Asendorf and Denissen 2006; Crockett et al. 2006; Laursen and Hoff 2006). With the person-centered approach, it is possible to identify individuals who score similar (who have the same profile) on a set of variables (like the four personality dimensions). Individuals with nearly identical profiles form a distinct subgroup or type. Different subgroups can be heterogeneous with respect to substance use (Laursen and Hoff 2006), which may provide important insights with respect to designing and tailoring interventions (e.g., Conrod et al. 2008; 2010).

As stated before, no person-centered typology of the SURPS has been conducted so far. One well-known person-centered typology in personality research is based on Block and Block’s (1980) constructs of ego-resiliency and ego-control, namely the resilients, undercontrollers, and overcontrollers (e.g., Dubas et al. 2002). In relation to the Big Five personality dimensions, high scores on emotional stability, extraversion, openness, agreeableness, and conscientiousness characterize the resilients. The undercontrollers show high scores on extraversion and moderate to low scores on emotional stability. Finally, the overcontrollers show low scores on extraversion, emotional stability, and openness (Knyazev and Slobodskaya 2006). Following this typology in relation to the SURPS personality dimensions, it is plausible to expect one group that is well adapted, one group that resembles the undercontrollers, and one group that resembles the overcontrollers. Considering that all four SURPS dimensions are risk traits for substance (mis)use, the well adapted group (resilients) will be characterized by the absence of these risk traits (i.e., low scores on all dimensions). Since behavioral undercontrol refers to the inability to inhibit behavior (e.g., Zucker et al. 2011) and extraversion is related positively to sensation seeking (Woicik et al. 2009), the group that resembles the undercontrollers will be high on sensation seeking and impulsivity and low on anxiety sensitivity and hopelessness. The overcontrollers, on the other hand, are low on emotional stability and extraversion and, therefore, will be high on anxiety sensitivity and hopelessness and low on sensation seeking and impulsivity (Knyazev and Slobodskaya 2006; Woicik et al. 2009).

In relation to substance use behaviors, personality traits concerning behavioral undercontrol (i.e., sensation seeking) relate to trajectories that show earlier onset, more consumption and greater persistence (Chassin et al. 2002; Hill et al. 2000) and personality traits concerning negative emotionality (i.e., hopelessness) are found to predict escalating trajectories of adolescent alcohol use (Chassin et al. 2002; Colder et al. 2002). Thus, behavioral undercontrol seems more relevant in relation to the onset of substance use, and negative emotionality in substance use maintenance. It might be, then, that adolescents with an undercontrolling typology are more at risk for an early onset of substance use behaviors than adolescents with an overcontrolling typology. In sum, integrating both approaches, while investigating the prospective role of the SURPS personality dimensions, can provide insights into how these personality dimensions explain variance not only in substance use (i.e., universal differences), but also in how group or individual differences in patterns of dimensions explain differences in substance use behaviors (i.e., individual differences).

## The Current Study

The present study integrates a person-centered approach with a variable-centered approach of the SURPS personality dimensions in relation to alcohol, tobacco, and marijuana use in early adolescence. With respect to the variable-centered analyses, we expect to find strongest effects for sensation seeking and hopelessness based on a prior study (Malmberg et al. 2010b). Specifically, we hypothesize that sensation seekers and individuals who report higher levels of hopelessness have an increased risk for early alcohol, tobacco, and marijuana use. Our main goal with respect to the person-centered analyses is to investigate whether different subgroups of individuals can be identified based on the personality dimensions. We hypothesize that three subgroups can be identified; one group that is low on all personality dimensions (resilients), one group with lower scores on anxiety sensitivity and hopelessness and higher scores on sensation seeking and impulsivity (externalizers), and a final group with higher scores on anxiety sensitivity and hopelessness and lower scores on sensation seeking and impulsivity (internalizers). In relation to substance use, we expect that having a resilient personality type will have a protective effect with respect to substance use behaviors in contrast to having an internalizing or externalizing personality type. We furthermore expect the externalizing adolescents to be more at risk for an early onset of alcohol, tobacco, and marijuana use, compared to the resilient and internalizing adolescents.

## Method

### Sample and Procedure

The data for this study were collected as parts of a broader effectiveness study on a national school prevention program “The Healthy School and Drugs” (Malmberg et al. 2010a). A total of 23 schools were included in the effectiveness trial from seven regions in The Netherlands. We visited participating secondary schools and during these visits we provided further information about the research project. In collaboration with the schools’ headmasters, we annually informed the students’ parents about the goals of the study by a letter in which parents also were explained they could refuse participation of their child in the study. Approval for the design and data collection procedures was obtained beforehand from the ethic committee of the Radboud University Nijmegen. The data for the first wave (T1) were collected between January and March 2009 and for the second wave (T2) between September and November 2010. At T1, all students in grade 9 (12–13 years) independently filled out a digital questionnaire during school hours in the presence of a teacher and a research assistant. The questionnaires were counterbalanced on alcohol, tobacco, and marijuana, thus six different versions were administrated. The exact same procedure was followed at T2. To overcome the possible interference of intervention effects, we only selected the data of the seven control schools for the present study.

At T1, a total of 1,259 ninth-grade students took part in the study of whom 61 (4.8 %) were absent (i.e., ill) during data-collection at T1 and 6 participants (0.5 %) were declined participation by their parents. To rule out possible effects that prior experiences with substance use might have on personality, we only selected the participants with no prior alcohol, tobacco, and marijuana experiences at baseline (*n* = 758). This sample included 356 boys (47 %). Of the 758 participants, positioned from lowest to highest educational level, a total of 7.7 % pursued preparatory vocational training (*n* = 58), 13.1 % pursued junior general secondary training (*n* = 99), 28.4 % pursued senior general secondary education (*n* = 215), 16.1 % pursued a combination of pre-university and senior general secondary education (*n* = 122), and 34.8 % pursued pre-university education (*n* = 264). The age of the participants ranged from 11 to 14 years (*M* = 12.88, SD = .41) at T1 and 97.1 % of the participants were of Dutch ethnic origin. At T2, a total of 648 students participated again (response rate 85.5 %) and 235 of these students reported drinking a glass of alcohol in the past (36.3 %), 128 students reported smoking (19.8 %), and 27 students reported marijuana use (4.2 %).

### Measures

#### Personality Dimensions

The personality dimensions were measured at T1 with the Dutch translation of the Substance Use Risk Profile Scale (SURPS: Woicik et al. 2009; Malmberg et al. 2010b). Factor structure, internal consistency and test–retest reliability, as well as construct, convergent, and discriminant validity of this instrument were shown to be good (Krank et al. 2011; Malmberg et al. 2010b; Woicik et al. 2009). The SURPS distinguishes four personality dimensions, namely anxiety sensitivity (i.e., the fear of physical arousal), hopelessness (i.e., negative thinking), sensation seeking (i.e., the urge for trying out new things), and impulsivity (i.e., difficulty in controlling behavioral responses). Each dimension was assessed using five to seven items that could be answered on a 4-point scale, ranging from 1 = “strongly agree” to 4 = “strongly disagree.” Example items are: “It’s frightening to feel dizzy or faint” for anxiety sensitivity, “I feel that I’m a failure” for hopelessness, “I like doing things that frighten me a little” for sensation seeking, and “I usually act without stopping to think” for impulsivity. Cronbach’s alphas were .67 for anxiety sensitivity, .76 for hopelessness, .66 for sensation seeking, and .63 for impulsivity. These reliability estimates converge with those from previous research (e.g., Jaffee and D’Zurilla 2009; Malmberg et al. 2010b) and are satisfactory for short scales (Loewenthal 1996).

#### Substance Use

We assessed adolescents’ alcohol use at T2 in terms of lifetime prevalence, which was measured by asking: “Have you ever drunk a glass of alcohol?” Participants could answer this question with yes (= 1) or no (= 0). Lifetime prevalence of tobacco use was also measured at T2 by a single item on a 9-point scale ranging from 1 = “I never smoked, not even a puff” to 9 = “I smoke at least once a day” (Kremers et al. 2001). To tap lifetime prevalence of smoking, adolescents who responded in the categories 2–9 were categorized as tried smoking before (= 1), and the adolescents who responded in category 1 were categorized as never tried smoking (= 0) following Kremers (2002). Finally, we assessed the lifetime prevalence of marijuana use at T2 through a single item, namely: “Have you ever used marijuana?” (Monshouwer et al. 2005). Participants could answer with yes (= 1) or no (= 0).

### Attrition Analyses

Of the 758 participants at T1, 648 were included again at T2. The participants lost to follow-up were compared with the remaining participants on the variables sex, age, education, and the SURPS dimensions using independent sample t-tests and Chi-square tests. Participants lost to follow-up were more likely to pursue preparatory vocational training or senior secondary training [χ^²^ (4, *n* = 758) = 27.15; *p* < 0.001]. No differences were found for sex, age, and the SURPS dimensions.

### Strategy of Analyses

First, we computed descriptive analyses of the personality dimensions (i.e., anxiety sensitivity, hopelessness, sensation seeking, and impulsivity) and alcohol, tobacco, and marijuana use, separately for sex and education. Second, to investigate whether participants’ sex and educational level should be specified as covariates in the model, we conducted two MANOVA’s to compare responses on the SURPS personality dimensions and substance use between males and females and between different educational levels. Post hoc tests with Bonferroni corrections were carried out to investigate the significant differences in education. Third, we determined the correlations between our model variables.

**Fig. 1:**
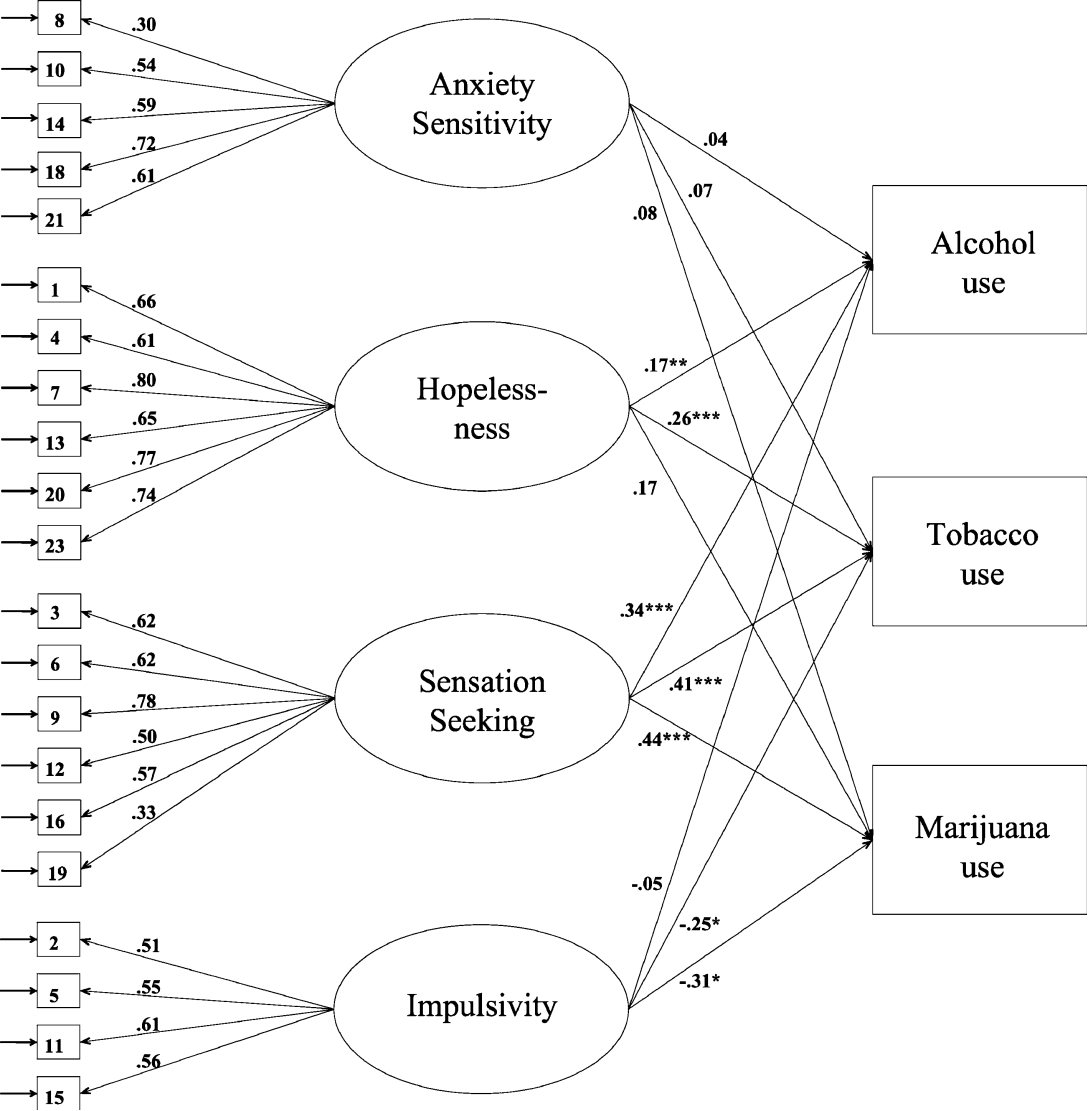
Standardized estimates of relationships between SURPS personality dimensions (T1) and substance use (20 months later) (*n* = 758). *Note.* Sex and education were specified as covariates. * p < .05, ** *p* < .01, *** *p* < .001

Then, in our variable-centered approach, we investigated the longitudinal relationships between the SURPS personality dimensions and lifetime prevalence’s by specifying and testing a first model (see Fig. 1) with structural equation modeling (SEM) in Mplus 6.1 (Muthén and Muthén 1998–2010). In these models, we included lifetime prevalence of alcohol, tobacco, and marijuana as observed variables and we added the personality dimensions as latent constructs, with separate scale items as indicators. Sex and education were specified as covariates in the model if the preceding MANOVA’s showed significant effects. The items of the personality dimensions have response categories varying from 1 to 4 and were treated as ordered categorical variables. We estimated the parameters in the model with probit regression using the Weighted Least Square with Mean- and Variance- adjusted Chi-square test statistic (WLSMV) estimator. The Chi-square and the *p* value, the Comparative Fit Index (CFI: Bentler 1989), and the Root Mean Square Error of Approximation (RMSEA: Steiger 1990) were used to assess the goodness of fit of the model (Hu and Bentler 1999). We used the explained variance as a measure of effect size (Cohen 1992). To correct for the multilevel structure of our data (i.e., data of individual students are nested within schools), we used the COMPLEX procedure in Mplus (cf Kuntsche and Jordan 2006; Malmberg et al. 2010b). To handle the problem of missing values, Mplus uses all available pairwise information in the data (Full Information Maximum Likelihood (FIML): Muthén and Muthén 1998–2010).

After that, in our person-centered approach, we performed Latent Profile Analyses (LPA). LPA is a special case of Latent Class Analysis (LCA). LCA is used with (un)ordered categorical as indicators of the latent classes, LPA with continuous indicators. We used the manifest scales (unstandardized scores) of the four personality dimensions at T1 to identify distinct profiles (subgroups) of personality in Mplus, using the Robust Maximum Likelihood estimator (MLR). In order to identify the most appropriate and parsimonious model, we examined one through five latent profiles by conducting a series of five nested models. The Bayesian Information Criterion (BIC; Schwartz 1978), the Bootstrap Likelihood Ratio Test (BLRT) and the Lo-Mendell-Rubin Likelihood Ratio Test (LMRT) have proven to be good and consistent statistical indicators in determining the most parsimonious profile solution in LCA models (Nylund et al. 2007). Profile sensitivities (the average profile-membership probability or posterior probability for each profile after classifying the participants in subgroups) and Entropy (an overall measure of all posterior probabilities) will be used as additional measures to decide which number of subgroups is appropriate. The BIC is used to asses model fit with lower BIC-values indicating a better fit, while the BLRT and LMRT p-values provided by the BLRT and LMRT can be used in order to test whether the model significantly improves after the inclusion of an additional profile (Nylund et al. 2007). Final determination of the number of profiles will also depend on other considerations like profile interpretability and distinctiveness, profile size, theoretical and scientific relevance.

Measurement invariance of the latent profiles will be examined for sex and for education using Multigroup Latent Profile Analysis (MLPA). After the final number of profiles is chosen, the unconstrained multigroup model is determined with profile sizes and means of the four personality dimensions allowing to vary free across sex (boys-girls) and education (5 levels) (using KNOWNCLASS in Mplus to define two or five classes respectively). The semi-constrained multigroup model will be tested with profile sizes allowed to vary but the means of the four personality dimensions constrained to be equal across sex or education. If the fit of the semi-constrained model will not significantly increase, measurement equivalence (equal means across sex or education) is supported. The fully constrained multigroup model will be tested by constraining the four personality means and profile sizes to be equal. If the fit of this model does not deviate significantly from the semi-constrained model, the profile sizes are not different across sex or education. The loglikelihood values and scaling correction factors of two subsequent models are used to compute a Chi-square difference test according to the steps as described on the website of Mplus (http://www.statmodel.com/chidiff.shtml).

Finally, we examined how the different personality types (profiles) relate to the lifetime prevalence of alcohol, tobacco, and marijuana use at T2. In Mplus, the three dependent variables were introduced as AUXILIARY variables. Then equality of means across the different personality profiles was tested with the Wald Chi-square test. Posterior probabilities are used as weight factors to account for profile membership uncertainty. First an overall test will be applied (are there possible significant differences between profiles for an auxiliary variable) before a posteriori testing differences between specific profiles.

## Results

### Descriptive Analyses

Table 1 presents the means and standard deviations of the SURPS’ personality dimensions and substance use, separately for sex and education. Two MANOVA’s were conducted to examine whether the personality dimensions and substance use significantly differed across sex and education (see Table 1). Girls scored significantly higher on anxiety sensitivity, whereas boys scored significantly higher on sensation seeking. Students who received junior general secondary training scored significantly higher on anxiety sensitivity compared to students who received preparatory vocational training. Students receiving general secondary training also scored higher on hopelessness and tobacco use compared to those receiving senior general secondary training or pre-university education. Finally, students receiving a senior general secondary training were more likely to smoke than students receiving a combination of senior general secondary training and pre-university education or solely pre-university education. Because of these significant effects of sex and education they were specified as covariates in the prospective analyses.

**Table 1:** Means and standard deviations for personality profiles and substance use, separately for sex and education

	Sex		Education					Total
	Female	Male	1.	2.	3.	4.	5.	
Personality profiles								
Anxiety sensitivity	2.44 (.62)*	2.23 (.64)*	2.15 (.70)^a^	2.47 (.62)^a^	2.30 (.62)	2.40 (.63)	2.34 (.65)	2.34 (.64)
Hopelessness	1.42 (.43)	1.34 (.38)	1.37 (.42)	1.52 (.45)^a^	1.44 (.42)^b^	1.38 (.40)	1.30 (.36)^ab^	1.39 (.41)
Sensation seeking	2.26 (.63)*	2.61 (.64)*	2.44 (.64)	2.44 (.62)	2.47 (.68)	2.30 (.65)	2.43 (.65)	2.42 (.66)
Impulsivity	2.22 (.62)	2.12 (.64)	2.33 (.71)	2.32 (.68)	2.22 (.58)	2.23 (.64)	2.14 (.63)	2.22 (.63)
Substance use								
Alcohol use	.38 (.49)	.36 (.48)	.39 (.50)	.39 (.49)	.38 (.49)	.30 (.46)	.38 (.49)	.37 (.48)
Tobacco use	.20 (.40)	.19 (.39)	.26 (.45)	.30 (.46)^ab^	.26 (.44)^cd^	.08 (.27)^ac^	.14 (.35)^bd^	.19 (.40)
Marijuana use	.03 (.18)	.05 (.21)	.05 (.23)	.03 (.16)	.08 (.27)^a^	.00 (.00)^a^	.03 (.17)	.04 (.20)

Education; 1 = preparatory vocational training, 2 = junior general secondary training, 3 = senior general secondary training, 4 = combination senior general secondary training and pre-university education, 5 = pre-university education

Means with the same superscripts are significantly different from each other. All at *p* < .05 with Bonferroni corrections for education

Pearson correlations (between personality dimensions), biserial correlations (personality dimensions with substance use variables) and tetrachoric correlations (between substance use variables) are presented in Table 2. Only impulsivity was positively related to the other personality dimensions. Thus, if students reported higher scores on impulsivity they also tended to score higher on anxiety sensitivity, hopelessness and sensation seeking. With respect to personality dimensions and substance use, sensation seeking was related to all of the substance use outcomes (i.e., alcohol, tobacco, and marijuana use). Further, significant relationships were present of hopelessness with alcohol and tobacco use. Anxiety sensitivity was not significantly correlated to substance use and impulsivity was positively correlated with alcohol use.

**Table 2:** Pearson, biserial, and tetrachoric correlations of personality dimensions (T1) and substance use (20 months later)

	1	2	3	4	5	6
1. Anxiety sensitivity	–					
2. Hopelessness	.05	–				
3. Sensation seeking	−.04	−.05	–			
4. Impulsivity	.12***	.17***	.34***	–		
5. Alcohol use	−.02	.10*	.23***	.13**	–	
6. Tobacco use	−.02	.14**	.23***	.05	.62***	–
7. Marijuana use	−.00	.05	.28***	−.01	.67***	.82***

* *p* < .05, ** *p* < .01, *** *p* < .001

### Personality Dimensions and Substance Use

The model as depicted in Fig. 1 (including covariances between the latent variables, but not shown here) showed a good fit to the data [χ^²^ (*df* = 263, *n* = 758) = 435, *p* < .001, RMSEA = .029, CFI = .928]. The model showed medium effect sizes for the relationships between the four personality dimensions and substance use; together with sex and education they explained 11.4 % of the variance in lifetime prevalence of alcohol use, 18.2 % of the variance in tobacco use, and 13.9 % of the variance in marijuana use. As can be seen in Fig. 1, standardized estimates for the effects of the personality dimensions on substance use revealed significant effects for hopelessness and sensation seeking on lifetime prevalence of alcohol use. These results indicate that youngsters with higher levels of hopelessness and sensation seeking were more likely to having used alcohol 20 months later. Further, we found similar effects of hopelessness and sensation seeking on lifetime prevalence of tobacco use. Adolescents who were high on hopelessness and sensation seeking were also more likely to having smoked 20 months later compared to adolescents who were low on these two dimensions. Finally, the analysis showed significant effects of sensation seeking on lifetime marijuana use. This means that adolescents who reported higher levels of sensation seeking had a higher chance of marijuana use 20 months later.

Although the analysis also indicated significant effects of impulsivity on tobacco use and marijuana use, these results are not interpretable due to a classical suppression effect concerning impulsivity (Tu et al. 2008). As can be seen from Table 2, impulsivity was not correlated to either tobacco or marijuana use. However, impulsivity was strongly related to sensation seeking, and further analyses showed suppression to take place when sensation seeking and impulsivity enter the model simultaneously. Therefore, we estimated two more models that are presented in Table 3; a first model to verify our theoretical expectations including anxiety sensitivity, hopelessness, and sensation seeking [χ^²^ (*df* = 183, *n* = 758) = 326.202, *p* < .001, RMSEA = .032, CFI = .92], and a second model to verify the non-existing relationship between impulsivity and tobacco and marijuana use including anxiety sensitivity, hopelessness and impulsivity [χ^²^ (*df* = 143, *n* = 758) = 310.309, *p* < .001, RMSEA = .039, CFI = .938]. As expected the first model revealed similar effects as the model that included all four personality dimensions; hopelessness to be indicative of alcohol and tobacco use and sensation seeking to be indicative of all three substances (see Table 3). Also, the second model confirmed the expectation of non-significant relationships between impulsivity and tobacco and marijuana use.

**Table 3:** Standardized estimates and *p* values for the two tested models

	Model 1						Model 2					
	Alcohol		Tobacco		Marijuana		Alcohol		Tobacco		Marijuana	
	Beta	*p*	Beta	*p*	Beta	*p*	Beta	*p*	Beta	*p*	Beta	*p*
Anxiety sensitivity	.032	.120	.008	.930	.010	.813	−.012	.567	.001	.994	.010	.804
Hopelessness	.153	< .001	.183	.005	.073	.627	.089	.140	.163	.023	.070	.680
Sensation seeking	.319	< .001	.267	< .001	.260	< .001	–	–	–	–	–	–
Impulsivity	–	–	–	–	–	–	.171	.030	.023	.681	−.033	.719

Sex and educational level were specified as covariates

### Latent Profile Analyses on the Personality Dimensions

We performed five subsequent LPAs to determine the most meaningful profiles based on the SURPS personality dimensions. Table 4 displays the values for the BIC, Entropy, LMRT, BLRT, profile size, and posterior probabilities for the one to five profile solutions. The BIC-value is increasing after the four-profile solution, the LMRT is non-significant in the four-profile solution and the BLRT is non-significant in the five-profile solution indicating that a four-, three-, and four-profile solution is preferred respectively. We further examined the three- and four-profile solutions on criteria like theoretical and scientific relevance, profile interpretability and profile distinctiveness. In both the three- and four-profile solutions, a group low on three personality dimensions and a mean value for anxiety, a group high on hopelessness and low on sensation seeking, and a group low on hopelessness and high on sensation seeking were found. Average levels on all personality dimensions characterized the fourth type in the four-profile solution, due to splintering of both the low hopelessness/high sensation seeking and the high hopelessness/low sensation seeking groups. Thus, we decided to further analyze the three-profile solution because it represented the theoretically hypothesized, most distinct typologies (1) resilients (45.3 %), (2) internalizers (22.4 %) and (3) externalizers (32.2 %). The entropy is rather low (.645), but the posterior probabilities have acceptable values (above .80).

**Table 4:** BIC values, entropy, LMRT and BLRT values for five latent profile models

	1 profile	2 profiles	3 profiles	4 profiles	5 profiles
Total					
BIC	5,240	5,076	5,016	4,968	4,989
Entropy		.831	.645	.720	.735
LMRT (*p* value)		191.3 (.000)	90.6 (.000)	78.5 (.071)	12.2 (.536)
BLRT (*p* value)		197.1 (.000)	93.2 (.000)	80.9 (.000)	12.6 (.192)
N_1_ (post. prob.)	753 (1.000)	602 (.967)	243 (.816)	310 (.838)	108 (.749)
N_2_ (post. prob.)		151 (.920)	341 (.821)	195 (.882)	195 (.891)
N_3_ (post. prob.)			169 (.879)	150 (.773)	16 (.740)
N_4_ (post. prob.)				98 (.854)	336 (.814)
N_5_ (post. prob.)					98 (.864)
Boys					
BIC	2,405	2,336	2,308	2,305	2,320
Entropy		.853	.704	.716	.758
LMRT (*p* value)		94.60 (.001)	55.51 (.004)	31.10 (.526)	13.73 (.234)
BLRT (*p* value)		97.82 (.000)	55.51 (.000)	32.17 (.000)	14.20 (.013)
N_1_ (post. prob.)	352 (1.000)	71 (.899)	66 (.909)	126 (.831)	106 (.804)
N_2_ (post. prob.)		281 (.970)	128 (.838)	86 (.860)	85 (.874)
N_3_ (post. prob.)			158 (.874)	108 (.826)	34 (.921)
N_4_ (post. prob.)				32 (.865)	126 (.833)
N_5_ (post. prob.)					1 (1.000)
Girls					
BIC	2,793	2,722	2,707	2,681	2,698
Entropy		.815	.604	.730	.740
LMRT (*p* value)		98.37 (.000)	43.06 (.114)	54.74 (.080)	12.35 (.082)
BLRT (*p* value)		101.66 (.000)	44.50 (.000)	56.56 (.000)	12.76 (.429)
N_1_ (post. prob.)	401 (1.000)	318 (.953)	99 (.851)	117 (.866)	118 (.866)
N_2_ (post. prob.)		83 (.917)	162 (.816)	50 (.910)	137 (.786)
N_3_ (post. prob.)			140 (.760)	159 (.818)	89 (.783)
N_4_ (post. prob.)				75 (.812)	9 (.853)
N_5_ (post. prob.)					48 (.925)

### Measurement Invariance of the Three Latent Profiles

Comparing the unconstrained model with the semi-constrained model for sex we found a difference in Chi-square of χ^²^(12) = 31.99 (*p* = .000) and comparing the semi-constrained model with the fully constrained model we also found Chi-square differences: χ^²^(2) = 49.30 (*p* = .000). This means that the three-profile solution shows differences across sex with respect to the mean personality dimensions and difference in prevalence. For education, we found χ^²^(16) = 12.0 (*p* = .743) for comparing the unconstrained model with the semi-constrained model and χ^²^(8) = 8.23 (*p* = .411) for comparing the semi-constrained model with the fully constrained model. Thus, no significant differences were found between the mean values of the personality dimensions across educational level and no significant differences in prevalence.

### Latent Profile Analyses for Boys and Girls

**Fig. 2:**
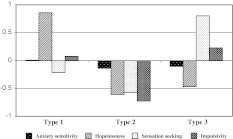
Standardized scores of the three types derived from boys’ reports of anxiety sensitivity, hopelessness, sensation seeking, and impulsivity

**Fig. 3:**
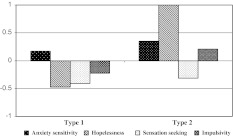
Standardized scores of the two types derived from girls’ reports of anxiety sensitivity, hopelessness, sensation seeking, and impulsivity

We repeated the procedure as described above for boys and girls separately, the results are shown in Table 4. For boys a four-profile solution (lowest BIC-value) or a three-profile solution (LMRT is non-significant for a four-profile solution) are possible. Again we found a profile high on hopelessness, a second profile low on three personality dimensions and a mean value on anxiety, and a profile high on sensation seeking (see Fig. 2). For the choice of a fourth profile, we have the same dilemma as mentioned before and decided to take a three-profile solution based on theoretical considerations (internalizers, 18.8 %; externalizers, 44.9 %: and resilients, 36.4 %). For girls, a two-profile solution is preferred (LMRT-value is non-significant for a three-profile solution, entropy value of .604 is very low for a three-profile solution. The two-profile solution is partly comparable with the solution for boys (see Fig. 3): one profile with low scores on three personality dimensions and a mean value on anxiety (resilients, 79.3 %) and one profile high on hopelessness (internalizers, 20.7 %). A profile with high levels of sensation seeking can not be found in the three to five profile solutions.

### Personality Profiles and Substance Use

In the final analyses, we tested whether the profiles showed significant differences with respect to alcohol, tobacco, and marijuana use at T2 (see Table 5). We found that the overall tests did not show significant differences for boys with respect to alcohol (χ^²^(2) = 2.83, *p* = .243), tobacco (χ^²^(2) = 4.92, *p* = .086) and marijuana (χ^²^(2) = 1.57, *p* = .457). For girls we also found no significant results with alcohol (χ²(1) = .89, *p* = .345), tobacco (χ^²^(1) = 1.08, *p* = .299), and marijuana (χ^²^(1) = .51, *p* = .473).

**Table 5:** Means and standard errors of the personality profiles (T1) on substance use (20 months later)

	Boys						Girls					
	Alcohol		Tobacco		Marijuana		Alcohol		Tobacco		Marijuana	
	M	SE	M	SE	M	SE	M	SE	M	SE	M	SE
Resilients	.30	.05	.14	.04	.03	.02	.35	.03	.18	.02	.03	.01
Internalizers	.38	.07	.25	.07	.06	.03	.42	.06	.24	.06	.05	.03
Externalizers	.40	.05	.24	.04	.07	.02	–	–	–	–	–	–

## Discussion

In samples with participants who already are using substances, it is well established that specific personality dimensions concerning neurotic tendencies and deficits in behavioral inhibition relate to substance (mis)use. The role of these personality characteristics in the onset of substance use in early adolescence is still mostly unclear. The present study represents one of the first to examine the predictive role of the four SURPS personality dimensions on the onset of substance use in early adolescence. In line with our expectations, the structural equation models showed that adolescents with higher levels of hopelessness and sensation seeking were more likely to start using alcohol and tobacco 20 months later. Also, sensation seekers were more likely ever to have used marijuana at follow-up. To come to a better understanding of processes and patterns underlying substance use behaviors in early adolescence, the present study combined this variable-centered approach with a person-centered approach. In the person-centered approach, individuals with similar profiles on the personality dimensions were identified. Our LPAs of the entire sample revealed three personality subtypes, namely resilients, internalizers, and externalizers. For boys and girls separately, the same personality subtypes were identified for boys, but only the resilient and internalizing subtypes were present for girls. Final analysis revealed no differences between the different personality profiles in relation to an early onset of substance use for both boys and girls.

### Personality Dimensions and Substance Use

In line with our expectations and prior work by Malmberg et al. 2010b, our longitudinal results indicate that hopelessness and sensation seeking seem most relevant for early substance use. The results with regard to sensation seeking are not unexpected given the novelty seeking nature of sensation seekers and that experimenting with different substances can be seen as such novel experiences. Hopelessness is often regarded as depression-proneness and individuals with more depressive symptoms generally show an increased risk for alcohol and tobacco use (e.g., Chaplin et al. 2009; Crum et al. 2008; Goodman and Capitman 2000). The role of hopelessness on early alcohol and tobacco use might be explained by a third variable explanation (e.g., early childhood problems), indicating that early childhood adversity can affect the development of personality dimensions, and subsequent engagement in problem behaviors (Akse et al. 2004; Hale et al. 2008; Malmberg et al. 2010b).

Surprisingly, impulsivity seems unrelated to beginning with substance use in early adolescence. Although previous studies suggest that impulsivity is an important predictor of substance use, we could not substantiate a significant role of impulsivity in our results. In the literature, impulsivity covers a wide range of definitions and concepts (Evenden 1999); dysfunctional impulsivity, motor and cognitive impulsiveness, and venturesomeness are just some of the many examples. Although impulsivity is a multidimensional construct, neurobiological theories suggest a two-factor model including reward seeking (i.e., sensation seeking) and disinhibition (i.e., unplanned behavior) to be most relevant for substance use behaviors (Dawe et al. 2004; Goldstein and Volkow 2002; Jentsch and Taylor 1999; Robinson and Berridge 2003). These theories suggest that the onset of substance use is related to increased dopaminergic activity in the mesolimbic reward system, and that substance use maintenance is related to a lack of inhibitory control (Flory and Manuck 2009). Our results are in line with this latter proposition, in that sensation seeking is relevant for substance use onset in contrast to impulsivity. Impulsivity, according to these theories, then would become important for subsequent substance use behaviors after use has started. This conclusion is also in line with scholarly arguments that impulsive individuals are more susceptible to the acute and rewarding effects of substances (Perkins et al. 2008), and are, therefore, more at risk for subsequent substance use behaviors after experiencing such rewarding effects at the start of use.

### Personality Profiles

Our main goal with respect to the person-centered analyses was to investigate whether different subgroups of individuals could be identified based on the four SURPS personality dimensions. In line with our hypotheses, we identified three personality subgroups for the entire sample: one group low on all personality dimensions (i.e., the resilients), one group high on hopelessness and low on sensation seeking (i.e., the internalizers), and a final group low on hopelessness and high on sensation seeking (i.e., the externalizers). It would be interesting for future research to investigate whether these different personality profiles also can be identified in other samples (e.g., different cultures, ages) and to disentangle what the relative roles of the different dimensions are in the identified personality subgroups. Are the identified subgroups mainly defined by one dimension or are the specific constellations between the different personality dimensions responsible for our findings?

The additional analysis indicated that different subgroups were present for boys and girls. Although the same three personality subgroups were identified for boys, only two of these subgroups were present for girls (i.e., resilients and internalizers). Overall, behavioral differences are present between boys and girls (e.g., Grant et al. 2004; Stinson et al. 2005), in that girls are more likely to report internalizing symptoms and boys are more likely to report externalizing symptoms (e.g., Angold et al. 2002; Hoffman and Su 1997; Wade et al. 2002). Given these behavioral sex differences, it seems plausible to find different subgroups for boys and girls. In concordance with the literature, we only found an externalizing subgroup for boys. However, an internalizing profile was present for both boys and girls, indicating a subgroup of boys with internalizing symptoms to be present in our sample. Since boys are believed to engage in more and more emotion-distracting behaviors during adolescence (Piko 2001), it would be interesting to examine how this particular subgroup evolves over time.

### Personality Profiles and Substance Use

In relation to substance use, we expected that a resilient personality profile would have a protective effect for early onset of substance use in contrast to having an internalizing or externalizing personality profile. Also, we expected externalizing adolescents to be at higher risk for an early onset of substance use, compared to resilient and internalizing adolescents. In contrast to these expectations, we did not find any differences between the different personality profiles for both boys and girls. This does not mean that the variable-centered results trump the person-centered results, since one explanation for the lack of findings might be the use of the posterior probabilities as weight factors in our analyses. Although the probabilities for substance use onset are in the expected direction, the use of the posterior probabilities increased the standard errors and subsequently lowered the possibility to find significant parameter estimates. Another explanation might be that the identified subgroups are not distinctive for the onset of substance use. However, it very well might be that the identified subgroups are distinctive for other substance related behaviors (e.g., escalation of use) or for other risk behaviors, like delinquency. It would be interesting to examine the (additional) value of the personality subtypes for different kinds of (substance use) behaviors and in other (already using) samples.

### Strengths, Limitations, and Implications for Future Research

A major strength of the present study is that it is one of the first to prospectively examine the role of the SURPS personality dimensions in early adolescence. Furthermore, we investigated this in a sample of early adolescents with no prior experience with alcohol, tobacco, and marijuana, thus without any interferences of substance use experiences. In doing so, we used well-validated measures with good psychometric properties. Finally, this study is the first to apply a person-centered approach on the SURPS personality dimensions and to identify different personality subtypes.

Besides these strengths, some limitations were present in the current study as well. First of all, our variable-centered analyses showed a classical suppression effect for impulsivity. One could argue that the four-factor model of the SURPS is less suited to study the start of substance use compared to substance use maintenance. However, it also could be that the suppression effect is due to sample characteristics. It might be that the suppression effect confines to the present sample, to samples of Dutch early adolescents, or to samples with no prior substance use experience. Future prospective research in early adolescence is necessary to clarify the origin of the suppression effect. A second limitation is that our use of self-reports might have lead to measurement errors, due to situational and cognitive influences (Brener et al. 2003). To overcome situational influences, like social desirability, and to optimize measurement validity, we guaranteed full confidentiality (anonymity) to our participants (e.g., Dolcini et al. 1996). To avoid cognitive influences (i.e., over or underestimations of substance use) we asked adolescents if they ever tried a specific substance, which one might expect participants to reliably recall. Thirdly, we solely focused on ever use of alcohol, tobacco, and marijuana use in early adolescence, without any prior experiences with these substances. Based on our findings, we might assume that the SURPS personality dimensions precede alcohol, tobacco, and marijuana use. However, it is still unclear whether experiences with these substances also modify personality traits and if potential changes due to substance use are noticeable. It seems likely that changes become more apparent after more exposure to substance use, but it also could be that only few experiences already influence the different personality traits. To deepen our knowledge on the bi-directional relationships between personality and substance use, it would be helpful to conduct cross-lagged analyses on the SURPS personality dimensions and substance use in future research. Finally, since we only used single item measures for our substance use outcomes in the current study (i.e., lifetime prevalences) it would be interesting to examine the role of the personality dimensions and profiles on a broader spectrum of substance use behaviors (i.e., quantity, frequency, and excessive drinking patterns). Also, in our outcome measures, we did not account for the potential role of parental permission. Future studies are necessary to investigate whether substance use with parental permission (e.g., a sip of wine at dinner) is conceptually different from substance use without parental permission in relation to the SURPS personality characteristics.

Overall, our results show that, in trying to prevent adolescents from alcohol, tobacco, and marijuana use at an early age, it may prove to be of key importance to focus on personality dimensions. This is especially relevant given the adverse health consequences of initiation of substance use in early adolescence, in combination with the fact that many adolescents start using substances in their early teens (Hibell et al. 2009; Monshouwer et al. 2008). Although recent preliminary evidence has shown that preventive intervention efforts may reduce adolescents’ risk behavior (Ozer et al. 2011), among which includes substance use, the present results indicate that significant gains can be achieved in clinical cost-effectiveness, by tailoring such prevention efforts–for example, in terms of intensity, duration, or specific methodology employed–to the exact needs of a subgroup based on their personality dimension.
